# Association of maternal nutrition with transient neonatal hyperinsulinism

**DOI:** 10.1371/journal.pone.0195383

**Published:** 2018-05-03

**Authors:** Mathilde Louvigne, Stephanie Rouleau, Emmanuelle Caldagues, Isabelle Souto, Yanis Montcho, Audrey Migraine Bouvagnet, Olivier Baud, Jean Claude Carel, Geraldine Gascoin, Regis Coutant

**Affiliations:** 1 Service de Diabétologie et Endocrinologie Pédiatrique, Departement de Pédiatrie, et Centre de Reference des Maladies Endocriniennes Rares de la Thyroïde et de l’Hypophyse, Centre Hospitalier Universitaire d’Angers, Angers, France; 2 Service de Pédiatrie, Centre Hospitalier du Mans, Le Mans, France; 3 Service de Réanimation et Médecine Néonatale, Centre Hospitalier Universitaire d’Angers, Angers, France; 4 Service de Diabétologie et Endocrinologie Pédiatrique, Service de Pédiatrie, Centre Hospitalier Universitaire de Nantes, Nantes, France; 5 Service de Réanimation et Médecine Néonatale, Centre Hospitalier du Mans, Le Mans, France; 6 Service de Néonatologie, Centre Hospitalier Universitaire de Nantes, Nantes, France; 7 Service de Réanimation et Pédiatrie Néonatale, Hôpital Universitaire Robert-Debré, Paris, France; 8 Service d'Endocrinologie Diabétologie Pédiatrique et Centre de Référence des Maladies Endocriniennes Rares de la Croissance, Hôpital Universitaire Robert-Debré, Université Paris Diderot, Sorbonne Paris Cité, AP-HP, Paris, France; Centre Hospitalier Universitaire Vaudois, FRANCE

## Abstract

**Objective:**

The objective was to determine whether maternal nutritional factors are associated with transient neonatal hyperinsulinism (HI).

**Design and setting:**

Case control study in 4 French tertiary Obstetrics and Neonatology Departments between 2008 and 2015.

**Methods:**

Sixty-seven mothers of neonates diagnosed with transient hyperinsulinism and 113 mothers of controls were included. The screening for hyperinsulinemic hypoglycemia in neonates was performed because of clinical symptoms suggestive of hypoglycemia or in the presence of conventional risk factors (small-for-gestational-age, prematurity, anoxo-ischemia, hypothermia, macrosomia, gestational diabetes). Hyperinsulinemic hypoglycemia was confirmed in the HI neonates and ruled out in the controls. This allowed for comparing maternal nutrition in cases and controls in a context of similar risk factors. One to 2 mothers of control neonates were included per case, and a food frequency questionnaire was addressed to the mothers between day 5 and day 10 after the birth of their newborn.

**Results:**

Crude odds ratio showed that maternal weight gain, abnormal fetal rate, C-section, gender, consumption of fresh cooked vegetables, fresh fruits and fruit juices, low fat diary products, light fat products, and daily bread were significantly associated with hyperinsulinism. Maternal body mass index, hypertension, gestational diabetes, birth weight percentile, gestational age and 5-minute Apgar score were not related to HI. In a multiple backward logistic regression model, consumption of fresh cooked vegetable ≥1/day (OR = 0.33 [0.14–0.77]) and light-fat products ≥1/week (OR = 0.24 [0.08–0.71]) was protective against hyperinsulinism, whereas gestational weight gain >20 kg (OR = 9.5 [2.0–45.5]) and between 15–20 kg (OR = 4.0 [1.2–14.0]), abnormal fetal heart rate (OR = 4.4 [1.6–12.0]), and C-section (OR = 3.4 [1.3–8.9]) were risk factors.

**Conclusions:**

A diet rich in fresh cooked vegetable and reduced in fat, together with the avoidance of a high gestational weight gain may be protective against transient neonatal hyperinsulinism.

## Introduction

Neonatal hyperinsulinism (HI) is the first cause of recurrent neonatal hypoglycemia [1]. The severe persistent form is most often of genetic etiology [2]. The incidence of minor and transient forms has been estimated to be 1/12000 births, four times more frequent than severe persistent forms, and this incidence is believed to be underestimated [3]. They usually recover spontaneously in several weeks [4].

The severity of neonatal HI results from neurological sequelae caused by neuroglycopenia and the associated suppression of lactates or ketone bodies, which cannot be used as alternative fuel to preserve neuronal function in the absence of glucose [2]. Indeed, neurological handicap has been observed in children with neonatal HI [5], with motor, language, and cognitive delay in 26 to 44% of cases, but also visual anomalies, convulsions or infantile spasms [2]. Although the outcome of transient HI was usually considered as benign compared to that of persistent HI,[4] some studies found that neurological consequences were similar in hypoglycemia due to transient and persistent neonatal HI [6, 7].

Because of the potential poor neurologic outcome, the identification of factors likely to be involved in the development of transient neonatal HI is of crucial importance. Conventional risk factors identified in transient forms of HI are prematurity, small weight for gestational age (SGA), antenatal and perinatal anoxo-ischemia, macrosomia, and gestational diabetes[2]. Transient HI is believed to result either from antenatal and/or perinatal stress, or from excess transplacental transfer of nutrients (such as glucose) with reactive fetal insulin overproduction [8]. More recently, maternal obesity, excessive maternal weight gain during pregnancy, and high carbohydrate intake have been associated with neonatal hypoglycemia, likely due to hyperinsulinism [9–11]. Several environmental or nutritional factors that are known to cause an increase in insulin production in the exposed subjects can also cross the placenta or be delivered to the fetus through a more complex placental metabolism. For instance, sweeteners or bisphenol A (a xeno-estrogen found in plastic bottles, microwavable ready meals and canned food and drinks) were all able to increase insulin secretion in animal models or in humans [12–14].

Overall, these studies suggested that maternal nutrition could be predictive of or protective against transient neonatal HI. To explore this hypothesis, we collected a food frequency questionnaire relative to dietary habits during pregnancy from 67 mothers of neonates diagnosed with transient neonatal HI, and compared to 113 mothers from neonates in whom HI was screened because of the presence of clinical symptoms suggestive of hypoglycemia or of conventional risk factors and excluded.

## Methods

This multicenter, case-control study was conducted in four Obstetrics and Neonatology Departments at Angers University Hospital, Nantes University Hospital, Robert Debré (Paris) University Hospital, and Le Mans General Hospital. All neonates were recruited because they had risk factors for hypoglycemia or presented clinical signs suggestive of hypoglycemia and all had blood glucose screening (see below). One hundred and twenty-three neonates less than 28 days-old were hospitalized between 01/01/2008 and 09/30/2015 with a diagnosis of hyperinsulinemic hypoglycemia. Subjects with monogenic (mutation in genes implicated in insulin secretion) or syndromic (Beckwith-Wiedemann syndrome) hyperinsulinism were excluded. For one mother of a neonate with hyperinsulinism, 1 to 2 mothers of neonates with no hypoglycemia were recruited.

### Detection of neonates with hyperinsulinemic hypoglycemia and selection of controls

Capillary blood glucose monitoring was performed in all neonates in the presence of clinical symptoms suggestive of hypoglycemia (irritability, tremulation, hypotonia, lethargy, apnea, tachypnea, poor feeding, hypothermia, or seizures), or in the presence of identified risk factors for hyperinsulinemic hypoglycemia (prematurity, small-for-gestational-age, antenatal or perinatal anoxo-ischemia, hypothermia, macrosomia, gestational diabetes). The capillary blood glucose monitoring protocol included a first measurement at clinical symptoms, or after the first feed at 4 hours of life, then before the second and third feed and finally every 12 hours. If capillary blood glucose was > 50 mg/dL (2.7 mmol/L) between 4 and 72 hours of life or > 60 mg/dL (3.3 mmol/L) thereafter, normoglycemia was retained and glucose monitoring stopped after the 5^th^ capillary blood glucose test. If capillary blood glucose was below these thresholds, a plasma blood glucose sample was taken and intensified capillary blood glucose monitoring undertaken, before every feed or at clinical signs. Hypoglycemia was defined by plasma glucose <30 mg/dL (1.7 mmol/L) between 4 and 48 hours of life which persisted after 48 hours or by plasma glucose <45 mg/dL (2.5 mmol/L) after 48 hours of life, as these two thresholds have been associated with later adverse cognitive outcomes [7, 15, 16, 17]. To avoid induced hyperinsulinism, the treatment of hypoglycemia was always initially conservative, and consisted in offering breast feeding more often under capillary blood glucose monitoring (to maintain capillary blood glucose > 30 mg/dl before 48 hours, and > 45 mg/dl thereafter), or offering formula for premature babies (which are slightly enriched in carbohydrates in comparison with classical formula). Neonatal HI was defined as plasma insulin concentration > 2 mIU/L during hypoglycemia and daily glucose needs > 10 mg/kg/min before the introduction of any specific treatments [2, 18]. When performed, a glucagon test (1 mg IM or IV) showing a glucose response > 30 mg/dl was considered indicative of hyperinsulinism [2, 18]. Transient hyperinsulinism was retained when daily glucose needs > 10 mg/kg/min and/or diazoxide treatment was necessary for at least 5 days, as the duration likely allowed for ruling out of transitional hypoglycemia, and could be stopped before 3 months [1,4]. Children from twin pregnancy and with gestational age < 34 weeks were not included. Control neonates had capillary blood glucose measurements in the same conditions as HI neonates, and hypoglycemia was ruled out. No venous puncture for plasma glucose and insulin measurements was performed in the controls.

### Data collection

The mothers were contacted by phone or directly between day 5 and day 10 following birth, and were asked to answer a questionnaire relative to food- and drink- frequency during pregnancy. The questionnaire was compiled from the MONA LISA-NUT study questionnaire and the ISIS-DIAB questionnaire [19, 20], both validated in French. It was short and qualitative to quickly assess the usual intake of French women during pregnancy. It contained 50 main questions, about foods commonly consumed in France. Items were grouped into the following macronutrient categories: starches (rice, bread, pasta, potatoes, etc.), animal-derived proteins (meat, fish, sea food, eggs, cured meat products such as ham and sausages), vegetables and fruits (fresh cooked vegetable, fresh fruits or fresh fruit juices, fruit juices with added sugar, 100% fruit juices, canned vegetable and canned fruits), dairy product (regular or low-fat), fast foods (pizza, hamburgers), microwavable ready meal, fat product (regular or light), sweets (sweet desserts, candies, chocolate bar, chocolate spread), sweeteners, soft drinks (regular, diet, canned), water (tap or bottled water), coffee and alcohol. Food consumption was divided into 5 categories: ≥ 1/day; ≥ 1/week, ≥ 1/month, < 1/month or never. The time taken to fill in the questionnaire ranged from 10 to 20 minutes.

Maternal and neonatal data were collected in the obstetrical and neonatal medical records. Total weight gain during pregnancy was recorded (difference between weight at the onset of labor, measured by the team in the Obstetrics Departments, and self-reported pre-pregnancy weight), as a reliable estimate of true caloric intake during pregnancy [21]. The mothers were screened for carbohydrates intolerance or gestational diabetes, as recommended [22, 23]. From 2007 to 2010, the pregnant women underwent a two-step screening process for gestational diabetes, as recommended by the American College of Obstetricians and Gynecologysts: first, a glucose challenge test was performed between 24 and 28 weeks of gestation followed by an oral glucose tolerance test if necessary [22]. From 2011, French recommendation changed for a one-step screening process using OGTT (75g) [23]. Mean annual household income, maternal age at birth, BMI at the onset of pregnancy, total weight gain during pregnancy, tobacco consumption, gestation number, and method of delivery were recorded. Newborn birth weight, length and head circumference, gender, 1- and 5-minute Apgar score, and gestational age (using ultrasonographic criteria from early ultrasound scans) were recorded.

### Statistics

The variables were presented as means ± SD, or medians (25^th^; 75^th^ percentiles). Comparisons between subjects were performed using the Student *t* test or Mann-Whitney U test. For discrete variables, significance was assessed using the Chi-squared test. The 5 initial categories were dichotomized at the cutoff closest to the 25^th^ or the 75^th^ percentile of controls, according to clinical relevance [24]. By doing so, we contrasted the upper (or the lower) quartile with the combination of the other quartiles. First, we examined the crude association of maternal food intake and maternal and fetal characteristics with transient congenital HI, and presented the odds ratio if p < 0.05. Five-minute Apgar score was used in the analyses since it is considered a better predictor of neonatal outcome as 1-minute Apgar score [25]. To select the best combination of predictors, we constructed a backward multiple logistic regression model with all variables significantly related to HI in simple logistic regression. Since our main objective was to study whether maternal nutrition could be predictive of or protective against transient neonatal HI, maternal BMI as well as gestational weight gain were kept in the final model. To compare the estimated to observed likelihood of transient congenital HI, the Hosmer-Lemeshow goodness-of-fit test was performed. All analyses were performed using the SPSS 19.0 package.

### Ethics

This study was approved by Angers University Hospital Ethics Committee, and written informed consent was collected from the parents.

## Results

**Fig 1:**
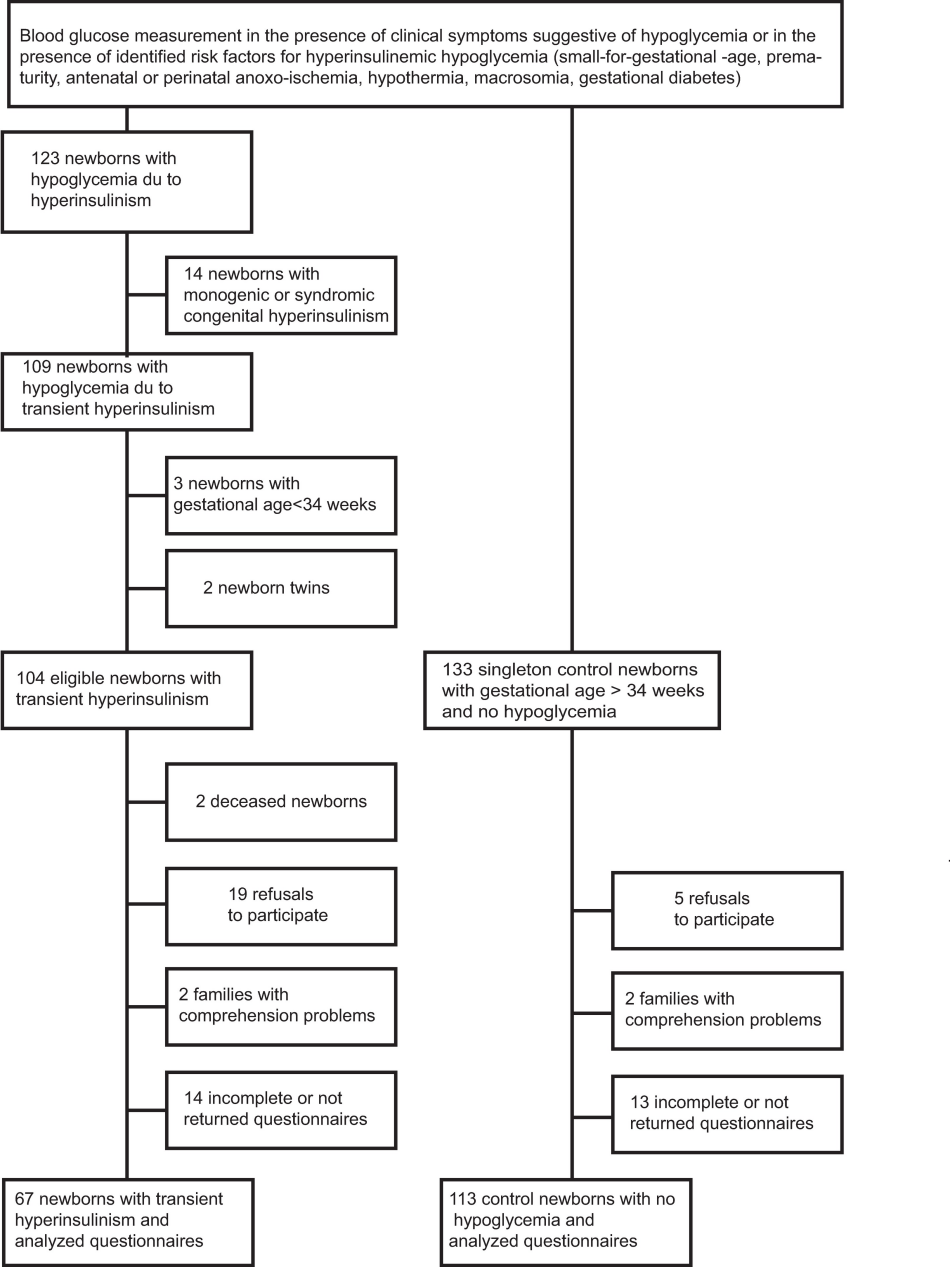
Flow chart of the HI and control groups. BMI: body mass index—HI: hyperinsulinism.

The study flow chart is shown in Fig 1. Data from 67 newborns with transient neonatal HI and 113 controls were analyzed. Twenty-three neonates with HI had a glucagon test (1 mg IM or IV) showing blood glucose response > 30 mg/dl following hypoglycemia. Thirty-three children received diazoxide treatment due to persistent hypoglycemia after a mean 11.5 ± 8.3 days, in order to decrease daily glucose needs. Maternal and neonatal characteristics are provided in Table 1. Maternal age, BMI, gestation number, gestational diabetes frequency, gestational age, birth weight percentiles (and percentages of small for gestational age and large for gestational age neonates), and 1- and 5-minutes Apgar score were similar in the HI and the control groups. The two groups were different for weight gain during pregnancy, rate of abnormal fetal heart rate, rate of cesarean section, and gender (p < 0.05).

**Table 1:** Maternal and neonatal characteristics for the HI and control groups.

	HI	Controls	p
N	67	113	
Maternal age, years	30.5 ± 5.4	30.1 ± 4.5	NS
BMI before pregnancy, kg/m^2^	23.4 ± 4.2	23.7 ± 5.7	NS
Mean Annual Household income, euros	41172 ± 18358	45472 ± 19309	NS
Gestation number	1.8 ± 1.1	1.6 ± 0.8	NS
Tobacco use during pregnancy	18 (27%)	27 (24%)	NS
Gestational weight gain			0.001
<10 kg	18 (27%)	33 (29%)	
10–15 kg	25 (37%)	60 (53%)	
15–20 kg	12 (18%)	16 (14%)	
> 20 kg	12 (18%)	4 (4%)	
Gestational diabetes	11 (16%)	19 (17%)	NS
Insulin treatment	4 (6%)	8 (7%)	NS
Regimen alone	7 (10%)	11 (10%)	NS
Hypertension	15 (22%)	11 (10%)	0.08
Abnormal fetal heart rate	35 (52%)	11 (10%)	<0.001
Cesarean section	37 (55%)	28 (25%)	<0.001
Gestational age^1^, wks	39.0 (37.0; 40.0)	39.3 (37.3; 40.3)	NS
Gender (M/F)	43/24	55/58	0.04
Birth weight, kg	2.70 ± 0.85	2.75 ± 0.63	NS
% of birth weight > 90^th^ perc	8	3	NS
% of birth weight < 10^th^ perc	50	41	NS
1-minute Apgar score^1^	9 (8; 10)	9 (8; 10)	NS
% of Apgar score < 5	10	10	NS
% of scalp pH < 7.0	7	4	NS
5-minute Apgar score^1^	10 (10; 10)	10 (10; 10)	NS
Plasma blood glucose, mmol/L	2.1 ± 1.0	*	
Plasma Insulin, IU/L	12.5 ± 18.4	*	

Mean ± SD and Student *t* test. HI: hyperinsulinism–BMI: body mass index–NS: non significant.

^1.^ Median (25^th^;75^th^ percentile) and Mann Whitney U test.

*Only capillary blood glucose was measured in controls: values were > 50 mg/dL [2.7 mmol/L] between 4 and 72 hours of life or > 60 mg/dL [3.3 mmol/L] thereafter.

There were significant differences between HI and control groups in consumption of fresh cooked vegetables, fresh fruits and fruit juices, low-fat dairy products, light fat-products, chocolate bar, and bread (S1 Table). The corresponding crude odds ratios for HI are shown in Table 2. Among maternal and fetal characteristics, maternal weight gain, abnormal fetal heart rate, C-section, and gender were also related to HI (Table 2).

**Table 2:** Crude odds ratio of explanatory variables for the occurrence of transient neonatal HI according to macronutrient consumption.

	Crude OR	95% CI
**Fresh cooked vegetable** ≥ 1/d	0.37^2^	0.20–0.70
**Fresh fruits—fruit juices** [≥1/wk]	0.34^1^	0.15–0.80
**Low-fat dairy product** ≥ 1/d	0.36^1^	0.16–0.80
**Daily bread consumption** (Yes)	0.42^1^	0.21–0.86
**Light fat-product** ≥ 1/wk	0.44^1^	0.22–0.88
**Maternal Weight Gain**		
<10 kg	1.43	0.68–3.00
10–15 kg	1	
15–20 kg	3.25^1^	1.24–8.50
>20 kg	8.13^3^	2.39–27.65
**Abnormal Fetal rate**	6.63^3^	3.25–13.54
**C-section**	3.75^3^	1.97–7.13
**Gender (M/F)**	1.89	1.02–3.51

HI: hyperinsulinism. Light fat products referred to products (used in cooking, spreads, and dressings) where the fat was lightened. Gestational age, maternal BMI, maternal hypertension, birth weight percentile, and 5-minutes Apgar score were not related to HI. CI: confidence interval; Bread consumption was for daily bread consumption at lunch and/or dinner.

^1^ p< 0.05

^2^ p < 0.01

^3^ p< 0.001

In a multiple backward logistic regression model including all significant maternal and fetal characteristics, and all significant dietary factors, consumption of fresh cooked vegetable ≥1/day (OR = 0.33 [0.14–0.77]) and light-fat products ≥1/week (OR = 0.24 [0.08–0.71]) was protective against HI, whereas gestational weight gain >20 kg (OR = 9.5 [2.0–45.5]) and between 15–20 kg (OR = 4.0 [1.2–14.0]), abnormal fetal heart rate (OR = 4.4 [1.6–12.0]), and C-section (OR = 3.4 [1.3–8.9]) were risk factors. Maternal body mass index, hypertension, gestational diabetes, birth weight percentile, gender, gestational age, and 5-minute Apgar score were not related to HI in the analyses (Table 3).

**Table 3:** Multiple backward logistic regression analyses for the occurrence of transient neonatal HI.

	OR	95% CI	p
**Fresh cooked vegetable** ≥ 1/d	0.33	0.14–0.77	<0.01
**Light fat-product** ≥ 1/wk	0.24	0.08–0.71	<0.01
**Weight gain during pregnancy**			
<10 kg	1.32	0.48–3.60	NS
10–15 kg	1		
15–20 kg	4.03	1.16–13.96	<0.05
>20kg	9.49	1.98–45.48	<0.01
**Abnormal fetal heart rate**	4.36	1.58–11.99	<0.01
**C-section**	3.39	1.30–8.87	< 0.05

HI: hyperinsulinism—OR: odds ratio–CI: confidence interval. Light fat products referred to products (used in cooking, spreads, and dressings) where the fat was lightened. Maternal hypertension, gestational age, 5-minute Apgar score, and birth weight percentile were not significantly related to HI, and were not entered in the multiple logistic regression analyses, whereas gender was no longer significant in the backward multiple logistic regression analyses. Maternal BMI, although non-significant, was forced as an adjusting variable (see methods). All the macronutrients that were associated (with p < 0.05) with neonatal HI were included in the multiple backward logistic regression analyses (see methods). The model correctly classified 84% of the subjects. Cox and Snell pseudo R square was 0.32. P value for Hosmer and Lemeshow goodness of fit was 0.28.

## Discussion

In this study, we found that mothers of newborns with transient HI compared to controls had significantly higher gestational weight gain, suggesting a higher caloric intake, and a “less health-conscious diet” in comparison to control mothers: they consumed fresh cooked vegetables and light-fat products significantly less frequently.

We found that light-fat products consumption provided protection from transient neonatal HI. This association was significant after adjustment for gestational weight gain, suggesting that it was independent of caloric intake. Fat from usual diet is typically of the saturated type. Animal studies have shown that the administration of a diet high in fat, especially saturated fat, during gestation led to increased plasma insulin and glucose levels in mothers, increased insulin secretion and lower plasma glucose in the pups, and increased plasma glucose and insulin in later life [26,27]. In humans, a high fat intake during pregnancy was associated with increased fasting insulin concentration after adjustment for BMI, however the effects on the neonates were not evaluated [28]. Our data may indicate that the preferential consumption of a diet reduced in fat in humans may protect from neonatal HI and hypoglycemia, as opposed to the effects observed in the offspring of animals fed a high-fat-diet.

We found that fresh cooked vegetable consumption provided protection against neonatal HI. In humans, lower vegetable fiber intake during pregnancy has been associated with an increase in plasma glucose and insulin, metabolic changes that were similar to those observed with a high fat intake [29]. No data were available regarding blood glucose and insulin in their neonates. No animal study has examined the effect of a diet rich in vegetables in pregnant mothers and in the pups. Although multiple confounding factors may exist, such as a more controlled caloric intake or other features of a “health-conscious diet”, fresh cooked vegetable consumption during pregnancy could provide protection against the development of hyperinsulinemia in newborns.

In this study, gestational weight gain over 15 kg was a risk factor for transient neonatal HI, independent of birth weight as well as 5-minute Apgar score. This was in agreement with studies that have shown that excessive gestational weight gain has been associated with a higher risk of hypoglycemia [10, 30]. In 2009, the American Institute of Medicine recommended the optimal gestational weight gain to be 11.5 to 16 kg if maternal BMI was between 19.8 and 26 kg/m^2^ [31]. Gestational weight gain is considered a reliable index of true caloric intake during pregnancy, likely better than food questionnaires, as most individuals underreport energy intake even during pregnancy [21, 32]. Moreover, it has been shown that excessive gestational weight gain occurred as a result of increased energy intake and not of a decreased energy expenditure and that this association was independent of BMI before pregnancy [33]. We therefore believe that the higher gestational weight gain in mothers of HI neonates reflected a higher caloric intake during pregnancy.

This study has some limitations. Firstly, the definition of transient HI could be questioned with respect to the so-called physiological transitional hypoglycemia. In transitional neonatal hypoglycemia, plasma glucose levels are low in the first hours of life and then progressively increase over 2 days to reach normal ranges [15]. The policy of the Obstetrics and Neonatology Departments participating in the study was to stabilize plasma glucose during the first 48 hours and to explore hypoglycemia which persisted after 48 hours of life [15, 34]. To avoid induced hyperinsulinism, the treatment of hypoglycemia was initially conservative, and consisted in offering breast feeding more often under capillary blood glucose monitoring (to maintain capillary blood glucose > 30 mg/dl before 48 hours, and > 45 mg/dl thereafter), or offering formula for premature babies (which are slightly enriched in carbohydrates in comparison with classical formula). In normal and at-term newborns, lower limits for plasma glucose have been established as 30 mg/dL after 4 hours and 45 mg/dL after 48 hours of life [15, 16]. Here, we only selected neonates whose hypoglycemia persisted after 48 hours of life and required treatment for at least 5 days, in order to rule out transitional hypoglycemia. Even if we cannot ascertain that transitional hypoglycemia was ruled out in all cases, it should be noted that our study also pointed to conventional factors of transient HI, such as perinatal stress (abnormal fetal heart rate), and cesarean section [2]. C-section has been shown to hamper hormonal transition in the neonate and favor hypoglycemia in some studies [35, 36]. Last, the reliability of the food frequency questionnaires may be brought into question [37]. Nevertheless the differences in food frequencies observed in this study were consistent, highlighting that mothers of newborns with transient HI ate less frequently a “health-conscious diet” during pregnancy. To reinforce these results our two populations were comparable for maternal BMI at the onset of pregnancy, maternal age, gestation number, gestational age, gestational diabetes and birth weight percentile, and the regression analyses were adjusted to maternal weight gain during pregnancy and BMI. Finally, mean annual household income was similar between the 2 groups, and in the range of the mean French household income [38].

In conclusion, our findings could reinforce the recommendations for optimal weight gain during pregnancy and also put forward some recommendations on macronutrient intake in order to reduce the risk of transient neonatal HI.

## Supporting information

S1 Table Frequency of food and drinks consumption in the HI and control groups. (DOC)

S1 File Excel data set. (XLSX)
