# Supplementary material for: Association of maternal nutrition with transient neonatal hyperinsulinism
Source: PLoS One. 2018 May 3;13(5):e0195383. doi: 10.1371/journal.pone.0195383 (PMC5933751; doi:10.1371/journal.pone.0195383)
Supplement: S1 Table — (DOC) [file pone.0195383.s001.doc]

**Supplementary Table: Frequency of food and drinks consumption** in the HI and control groups

|  | **HI** | **Controls** | **p** |
| --- | --- | --- | --- |
| Meat [<1/d / ≥1/d](%) | 39/61 | 43/57 | NS |
| Fish [<1/wk / ≥1/wk](%) | 36/64 | 47/53 | NS |
| Sea food [never / sometimes](%) | 47/53 | 38/62 | NS |
| Eggs [<1/wk / ≥1/wk](%) | 43/57 | 45/55 | NS |
| Cured meat products [<1/wk / ≥1/wk](%) | 45/55 | 37/63 | NS |
| Fresh cooked vegetable [<1/day / ≥1/d](%) | 62/38 | 37/63 | <0.01 |
| Canned vegetable [<1/wk / ≥1/wk](%) | 37/63 | 45/55 | NS |
| Fresh fruits and/or fresh fruit juices [<1/wk / ≥1/wk](%) | 24/76 | 10/90 | < 0.01 |
| Fruit juices with added sugar [<1/wk / ≥1/wk](%) | 63/37 | 63/37 | NS |
| 100% fruit juices [1/wk / ≥1/wk](%) | 69/31 | 69/31 | NS |
| Regular dairy product [<1/d / ≥1/d](%) | 13/87 | 21/79 | NS |
| Low-fat dairy product [<1/d / ≥1/d](%) | 87/13 | 70/30 | <0.05 |
| Microwavable meal [<1/wk /≥1/wk](%) | 73/27 | 74/26 | NS |
| Starches [<1/d / ≥1/d](%) | 27/73 | 24/76 | NS |
| Fast-foods [<1/month / ≥1/month](%) | 80/20 | 79/21 | NS |
| Daily bread consumption at lunch or dinner (%) | 67 | 83 | <0.05 |
| Light fat-product [<1/wk / ≥1/wk](%) | 79/21 | 62/38 | <0.05 |
| Regular fat-product [<1/d / ≥1/d](%) | 23/77 | 29/71 | NS |
| Sweet desserts [<1/wk / ≥1/wk](%) | 15/85 | 18/82 | NS |
| Candies [<1/wk / ≥1/wk](%) | 72/28 | 67/33 | NS |
| Chocolate bar [<1/wk / ≥1/wk](%) | 33/67 | 47/53 | 0.08 |
| Chocolate spread [<1/wk / ≥1/wk](%) | 75/25 | 66/34 | NS |
| Sweeteners [never/ yes](%) | 85/15 | 84/16 | NS |
| Light soft drinks [<1/wk / ≥ 1/wk](%) | 90/10 | 80/20 | 0.10 |
| Regular soft drinks [<1/wk / ≥1/wk](%) | 61/39 | 59/41 | NS |
| Coffee [1/d / ≥1/d](%) | 48/52 | 58/42 | NS |
| Alcohol [never / yes](%) | 85/15 | 88/12 | NS |
| Tap water (%) | 64 | 74 | NS |
| Bottled water (%) | 61 | 62 | NS |

HI: hyperinsulinism – wk: week – d: day – NS: non significant. All p values <0.10 were indicated
